# From Gene to Protein: Unraveling the Reproductive Blueprint of Male Grey Squirrels via Nerve Growth Factor (NGF) and Cognate Receptors

**DOI:** 10.3390/ani14223318

**Published:** 2024-11-18

**Authors:** Francesca Mercati, Gabriella Guelfi, Antonello Bufalari, Cecilia Dall’Aglio, Chiara Suvieri, Paolo Cocci, Francesco Alessandro Palermo, Polina Anipchenko, Camilla Capaccia, Beniamino Cenci-Goga, Massimo Zerani, Margherita Maranesi

**Affiliations:** 1 Department of Veterinary Medicine, University of Perugia, Via San Costanzo 4, 06126 Perugia, Italy; francesca.mercati@unipg.it (F.M.); gabriella.guelfi@unipg.it (G.G.); apsvet93@gmail.com (P.A.); camilla.capaccia@dottorandi.unipg.it (C.C.); beniamino.cencigoga@unipg.it (B.C.-G.); massmo.zerani@unipg.it (M.Z.); margherita.maranesi@unipg.it (M.M.); 2 Department of Medicine and Surgery, University of Perugia, Piazzale Settimio Gambuli, 1, 06129 Perugia, Italy; chiara.suvieri@unipg.it; 3 School of Bioscience and Veterinary Medicine, University of Camerino, Via Gentile III Da Varano, 62032 Camerino, Italy; paolo.cocci@unicam.it (P.C.); francesco.palermo@unicam.it (F.A.P.)

**Keywords:** grey squirrel, NGF, P75NTR, NTRK1, testis, reproductive phases

## Abstract

The grey squirrel, an invasive species, threatens the Eurasian red squirrel’s conservation, particularly in Umbria, Italy. Understanding its reproductive biology is essential to limiting its reproductive success. This study investigates the NGF system and its receptors (NTRK1 and p75NTR) in the testes of male grey squirrels, following prior research on female reproductive biology. NGF plays a role in testicular morphogenesis and spermiogenesis in animals and humans. As part of the LIFE Project U-SAVEREDS, eighteen squirrels were captured and classified into three morphotypes (immature, pubertal, and active spermatogenesis). NGF and its receptors were analyzed using real-time PCR, western blotting, immunohistochemistry, and plasma levels measured via ELISA. NGF qPCR expression levels were significantly higher during puberty compared to the immature and spermatogenesis stages (*p* < 0.01). Immunohistochemistry revealed NGF in Leydig cells, with stronger staining in pubertal and mature squirrels, while NTRK1 was found in Leydig cells in immature squirrels and germ cells in pubertal and mature ones. NGF receptors were observed in Sertoli cells in pubertal and mature squirrels. Plasma NGF levels showed a significant upregulation in pubertal squirrels (135.80 ± 12 pg/mL) compared to those in the immature (25.60 ± 9.32 pg/mL) and spermatogenesis stages (34.20 ± 6.06 pg/mL), with a *p* value < 0.01. The co-localization of NGF and its receptors suggests that NGF, produced by Leydig cells, regulates testis development and reproductive success through autocrine or paracrine mechanisms, potentially involving an unidentified pathway.

## Simple Summary

The grey squirrel (*Sciurus carolinensis*) is an invasive species threatening the Eurasian red squirrel in Umbria, Italy. Understanding its reproductive biology is crucial for managing its population. Based on previous research on female grey squirrels, this study investigates the nerve growth factor (NGF) system and its receptors in the testes of male grey squirrels. Eighteen squirrels were classified into immature, pubertal, and active spermatogenesis. Results showed an increased NGF level in pubertal squirrels. Immunohistochemistry identified NGF in Leydig cells, with stronger staining in pubertal and mature subjects. NTRK1 was found in the Leydig cells of immature squirrels and the germ cells of pubertal and mature ones. NGF receptors were also observed in Sertoli cells. These findings suggest that NGF plays a key role in testis development and reproductive success through autocrine or paracrine mechanisms, highlighting its importance in managing this invasive species.

## 1. Introduction

Initially, the function of NGF (nerve growth factor) was thought to be limited to the growth of the nervous system and neuron differentiation and survival [1,2]. However, the discovery of NGF in semen [3,4] and the presence of a potent ovulation-inducing neurotrophin in seminal plasma [5] shifted research toward the role of NGF in the female reproductive endocrine system. These findings provided the basis for hypothesizing that seminal plasma may play a role in female ovulation, which was also suggested by several recent studies showing that NGF was directly implicated in male and female reproductive functions [6,7,8,9,10]. More specifically, the expression of NGF and its receptors was evaluated in the testes of several mammalian species, including mice, llamas, cattle, bison, elk, white-tail deer, rats, and rabbits [6,11,12], underscoring the essential autocrine and/or paracrine role of NGF in the development of spermatozoa [13,14]. In wild male ground squirrel testes, Zhang et al. (2015) identified NGF-, NTRK1-, and p75NTR-signaling during seasonal spermatogenesis [15].

NGF was the first member of the neurotrophin family discovered. The initial evidence for the presence of this protein dates back to the early 1950s, when Rita Levi-Montalcini identified it in mouse sarcoma cultures [16]. The biological function of NGF is mediated by neurotrophic receptor tyrosine kinase 1 (NTRK type 1) [17], with high NGF affinity, and p75 neurotrophin receptor (p75NTR), with low NGF affinity [18]. The tropomyosin receptor kinase (TRK) family of receptor tyrosine kinases are encoded by NTRK genes; a family of three proto-oncogenes including neurotrophin *TrkA*, *TrkB*, and *TrkC*, which encode the NTRK1, NTRK2, and NTRK3 protein receptors. TRKB binds brain-derived neurotrophic factor (BDNF) and neurotrophins NT-4 and NT-5, and TRKC binds the neurotrophin NT-3. In addition to binding with low-affinity NGF, the p75NTR binds with the neurotrophin p75.

On the neuronal plasmatic membrane, the binding of NGF/NTRK1 triggers NTRK1 dimerization, autophosphorylation, and activation. The NGF/NTRK1 complex is then internalized into the cell via endocytosis by signaling endosome formation [19]. Inside the cell, NGF/NTRK1 induces the activation of the NGF-dependent transcriptional program through critical signaling cascades that mediate the transcription of NGF-regulated genes [20]. NGF and its receptors are dynamically regulated in response to physiological cellular events, such as cell growth and differentiation, and pathological events, such as tissue damage and diseases of the immune and inflammatory system [21].

The eastern grey squirrel (*Sciurus carolinensis*) is a medium-sized tree squirrel within the Sciuridae family and the order Rodentia, native to eastern North America. Males and females show no size or coat color differences [22]. Recognized as a major pest in Europe and globally, this species is listed among the 100 most invasive alien species (IAS) by the International Union for Conservation of Nature [23]. To counter the spread of IAS, the European Union implemented Regulation 2014/1143, aimed at preventing and controlling their introduction. In the UK and Italy, grey squirrels from the USA have displaced native red squirrels (*Sciurus vulgaris*), exemplifying competitive exclusion by an introduced species [24,25,26].

Maranesi et al., 2020 [27], observed that the Umbrian male grey squirrel has different testis morphotypes belonging to different reproductive phases: immature, pubertal, and active spermatogenesis (sexually mature). Increasing evidence underscores the need for a deeper understanding of the reproductive system of this invasive species, given its remarkable adaptability to new environments and its higher reproductive success compared to native species [27,28]. This study investigates the reproductive system of male grey squirrels across different developmental phases (immature, pubertal, and active spermatogenesis) with a focus on the role of NGF and its receptors, NTRK1 and p75NTR. Understanding these mechanisms provides critical insights into reproductive adaptations in invasive species, with implications for population management strategies. We examined the expression of NGF, NTRK1, and p75NTR at both gene and protein levels, along with the cellular localization of these molecules within the testes, to elucidate their roles in NGF signaling pathways across different reproductive stages. To complement this study, plasma NGF protein was also evaluated.

## 2. Materials and Methods

### 2.1. Animal Capture and Sample Collection

This study is part of the LIFE U-SAVEREDS Project (LIFE13 BIO/IT/000204) Management of grey squirrel in Umbria: conservation of red squirrel and preventing loss of biodiversity in the Apennines, investigating the conservation of the European red squirrel and forest ecosystem in Umbria and central Italy [29]. Between 2016 and 2018, as part of a control initiative, several male grey squirrels of three distinct morphotypes (*n* = 5 immature, *n* = 3 pubertal, and *n* = 10 active spermatogenesis; Maranesi et al., 2020) were captured using Tomahawk live traps (model 202.5, Tomahawk Live Trap Co., Hazelhurst, WI, USA). These traps were placed in shaded locations and away from high-traffic areas to reduce stress in the animals [27]. All captures complied with wildlife control regulations under Italian Law 157/92 (Rules for the protection of wild animals and homeotherms and hunting), the Habitat Directive 92/43/Comunità Economica Europea, and Regulation 2014/1143 of the European Parliament (focused on invasive alien species prevention and management). The captured squirrels subsequently underwent surgical gonadectomy [27] for testis collection.

Within a few minutes, to investigate the gene expression and western blot analysis, the testes were washed in an RNase-free phosphate-buffered saline solution and then frozen at −80 °C [30]. Testis samples for histological evaluation were quickly dipped in 10% neutral-buffered formalin solution in phosphate-buffered saline (PBS 0.1 M, pH 7.4) and, after 36 h, processed until paraffin wax embedding [27]. Blood samples (1 mL) were drawn from the radial vein of each anesthetized squirrel immediately before surgery. Plasma was separated by centrifuging the samples at 1500× *g* for 10 min, then stored at −20 °C for subsequent NGF analysis [31].

### 2.2. Histological Germinal Functional Phase Evaluation

Sections each five micrometers thick, were prepared, placed onto poly-L-lysine-coated glass slides, and allowed to air dry at 37 °C. The sections were subsequently stained with Haematoxylin–Eosin to histologically evaluate the functional phase of the germinal epithelium by using a photomicroscope (Nikon Eclipse E800, Nikon Corp., Tokyo, Japan) connected to a digital camera (Nikon Dxm 1200 digital camera, Tokyo, Japan) [32].

### 2.3. RNA Extraction from FFPE Tissues and qPCR Gene Expression

Total RNA was isolated from testicular tissue as described previously [33]. Five µg of RNA were reverse transcribed into 20 µL of iSCRIPT cDNA using random hexamers, following the manufacturer’s protocol (Bio-Rad Laboratories, Milan, Italy). Genomic DNA contamination was assessed by conducting a PCR reaction in the absence of reverse transcriptase. A series of experiments were performed to optimize quantitative reaction conditions, including efficiency and Cq (quantification cycle) values. The final optimized qPCR reaction volume of 25 µL contained 12.5 µL of iQSYBR Green SuperMix (Bio-Rad Laboratories), 1 µL each of forward and reverse primers (10 µM stock concentration), and water up to 25 µL. The primer sequences used are provided in Table 1.

All reagents were combined into a master mix and aliquoted into a 96-well PCR plate, followed by the addition of 2 µL of diluted cDNA (1:10). PCR was conducted on an iCycleriQ system (Bio-Rad Laboratories) with an initial denaturation at 95 °C for 1.5 min, then 40 cycles of 95 °C for 15 s and 53 °C for 30 s. A melting curve analysis was performed post-PCR to assess primer specificity using a protocol of 80 heating cycles for 10 s each, beginning at 55 °C with 0.5 °C increments. The melting curve confirmed the presence of a single amplification product for each primer set, validating primer specificity. Quantitative PCR values were normalized using the 2^−ΔCq^ method [34], with *Actin B* (*ACTB*) serving as the reference gene.

### 2.4. Western Blotting Protein Expression

The expression of NGF proteins was analyzed by western blotting in the testis tissue of immature (*n* = 3), pubertal (*n* = 3), and active spermatogenesis (*n* = 5) squirrels. Proteins were purified from testicular tissue homogenized in 1 mL of ice-cold RIPA buffer as previously described [28]. Following a 60 min incubation at 4 °C, the homogenates were centrifuged at 12,000× *g* for 20 min at 4 °C. Protein concentration in each supernatant was determined using the Bradford assay (Bio-Rad, Hercules, CA, USA). Equal protein amounts (50 μg) were then separated on a 10% SDS-PAGE gel (sodium dodecyl sulfate polyacrylamide gel electrophoresis 4% stacking gel, run for 1 h at 200 V and 500 mA). Proteins were subsequently transferred onto nitrocellulose membrane using the Trans-Blot Turbo Transfer System (Bio-Rad, Hercules, CA, USA). Next, the membrane was blocked in TBS with Tween-20 and 5% non-fat dried milk. The transferred proteins were probed with different antibodies, each one incubated overnight at 4 °C in separate western blots with rabbit anti-NGF (1:500; *n*. MA5-32067 Invitrogen, Waltham, MA, USA), rabbit anti-p75 (1:1000; ab52987, Abcam, Cambridge, UK), rabbit anti-pan-TRK (1:1000; *n*. ab109010 Abcam), and mouse anti-Actin (1:1000; clone AC-40, Merk Sigma-Aldrich, Darmstadt, Germany) antibodies. The pan-TRK antibody recognized the active domains of the neurotrophins NGF, BDNF, and NT-3. Then, western blots were incubated with a mouse horseradish peroxidase (HRP)-labeled secondary antibody (1:10,000; Merk Millipore, Burlington, MA, USA) for 1 h at room temperature under gentle agitation. Antibody incubations were performed in TBS. The immune-blotting substrates were detected by enhanced chemiluminescence (ECL, Bio-Rad, Hercules, CA, USA) with a ChemiDoc MP Imagin System (Bio-Rad Laboratories, Milan, Italy). For each target, densitometric analysis of specific signals was performed with ImageLab software version 6 (Bio-Rad Laboratories, Milan, Italy), and samples were normalized as a ratio to corresponding actin band intensity and used as a loading control.

### 2.5. Immunohistochemistry Protein Localization

Immunohistochemistry was conducted on samples from all morphotypes as described previously [33]. Sections of formalin-fixed, paraffin-embedded (FFPE) tissue, approximately 5 µm thick, were cut and mounted on poly-L-lysine-coated glass slides, then deparaffinized in xylene and rehydrated through graded ethanol to distilled water. Antigen retrieval was achieved by microwaving the sections in citrate buffer (pH 6.0) for three cycles of 5 min each at 750 W. Endogenous peroxidase activity was inhibited by treating the sections with a 3% hydrogen peroxide solution for 10 min. Non-specific backgrounds were avoided with a 30 min use of a species-specific normal serum diluted 1:10. For the immunohistochemical reaction, sections were incubated overnight at room temperature with a primary antibody (Table 2).

The optimal dilution for each primary antibody, chosen to maximize signal intensity while minimizing background, was identified from a range of 1:50 to 1:1000. On the second day, sections were incubated with a biotin-conjugated secondary antibody diluted 1:200 (Table 2). Immunoreactivity was visualized using an avidin–biotin complex (Vectastain Elite ABC Kit, Vector Laboratories, Burlingame, CA, USA) and developed with chromogen diaminobenzidine (DAB Substrate Kit, Vector Laboratories, Burlingame, CA, USA). Negative control sections were treated with normal IgG (Novus Biological, Littleton, CO, USA; Table 2) instead of the primary antibody. Since the molecules investigated in this study have already been identified in the ovary of the grey squirrel, ovarian sections of this species served as positive controls [31].

The sections were washed with PBS between all incubation steps, except after normal serum. All steps after endogenous peroxidase blocking were carried out in a moist chamber at room temperature. Sections were observed under a photomicroscope (Nikon Eclipse E800, Nikon Corp., Tokyo, Japan) connected to a digital camera (Nikon Dxm 1200 digital camera).

### 2.6. ELISA NGF Plasma Levels

An enzyme-linked immunosorbent assay (ELISA) was used to determine NGF levels in plasma samples (catalog number DY256, DuoSet ELISA for measuring NGF—R&D System, Milan, Italy), according to the manufacturer’s instructions. This kit had already been validated for the detection of NGF in grey squirrels (*Sciurus carolinensis*) [31]. Briefly, the plates were pre-coated with a diluted capture antibody at room temperature. After three washes, plates were blocked by adding reagent diluent to each well. Subsequently, 100 µL of each sample or NGF standard was added to the plate and incubated for 2 h at room temperature. Following incubation, a specific detection antibody and streptavidin-horseradish peroxidase (HRP) were added to each well, after which a substrate solution was applied and the plate was incubated for an additional 30 min to allow color development. The optical density of each well was then measured immediately, with readings set at 450–570 nm.

### 2.7. Data Statistical Analysis

All statistical analyses were performed using Prism version 10 (GraphPad Software Inc., San Diego, CA, USA). Data are expressed as mean ± SD (standard deviation) from at least three independent experiments. Statistical significance was set at *p* < 0.05. Analyses were conducted using one-way ANOVA (Analysis of Variance), followed by the Newman–Keuls Multiple Comparison Test.

## 3. Results

### 3.1. Histological Evaluation of the Testis Reveals the Phase of the Reproductive Cycle

**Figure 1:**
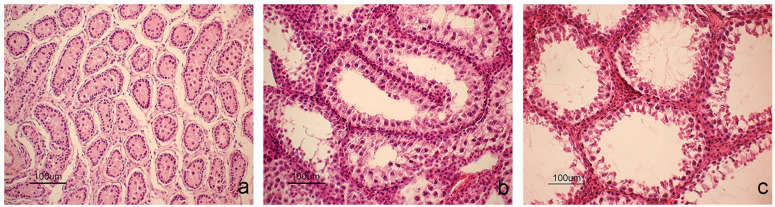
Histological evaluation of the squirrel testis. In the immature phase (**a**), the seminiferous tubules lack lumens and have small diameters. Sertoli cells are located at the basis of the germinal epithelium, while a few spermatogonia occupy a more central position. During the pubertal phase (**b**), the lumen starts forming in some seminiferous tubules. The germinal epithelium contains Sertoli cells, primary spermatocytes, and spermatids, with Leydig cells seen in the peritubular interstice. In the spermatogenesis phase (**c**), the seminiferous tubules have large lumens, and elongated spermatids are visible.

Histological analysis identified specific germinal epithelium features that determine the reproductive status of a squirrel [27]. The squirrels were categorized as immature (*n* = 5), pubertal (*n* = 3), and active spermatogenesis (*n* = 10). Immature squirrels (Figure 1a) showed seminiferous tubules lacking a lumen and of small diameter, with the germinal epithelium consisting of Sertoli cells close to the basal lamina and a few spermatogonia. Pubertal squirrels exhibited a germinal epithelium (Figure 1b) containing basal Sertoli cells and primary spermatocytes, progressing up to round spermatids, but without elongated spermatids or spermatozoa. The seminiferous tubules of pubertal squirrels were larger in diameter with a central lumen. Mature, sexually active male squirrels (Figure 1c) had testes with large seminiferous tubules and wide lumens, where spermatids and spermatozoa were in the germinal epithelium. Leydig cells in the intertubular connective tissue were observed to have abundant cytoplasm.

### 3.2. QPCR Gene Expression Levels of NGF and NGF Receptors

**Figure 2:**
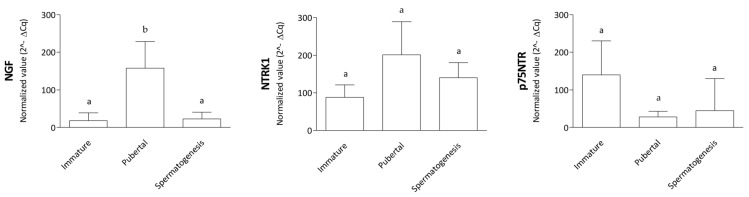
Gene expression-normalized data. The figure shows NGF, NTRK1, and p75NTR qPCR expression data normalized to the reference gene *ACTB*. Gene expression is compared between the three reproductive stages: immature, pubertal, and spermatogenesis. The expression levels of the *NGF* gene are different in the pubertal group versus the immature and spermatogenesis groups (*p* < 0.01). Different letters placed on the top of the boxes indicate statistically significant differences.

Normalized qPCR data revealed an NGF expression upregulated in the pubertal versus immature and spermatogenesis (*p* ˂ 0.01) phases. In contrast, an absence of differences (*p* > 0.05) was observed in NTRK1 and p75NTR expression levels examined in the three reproductive phases (Figure 2).

### 3.3. Western Blotting Protein Expression of NGF and NGF Receptors

Western blot analysis of grey squirrel testes revealed the expression of NGF, pan-NTRK, and p75NTR proteins during the three reproductive stages: immature, pubertal, and spermatogenesis.

**Figure 3:**
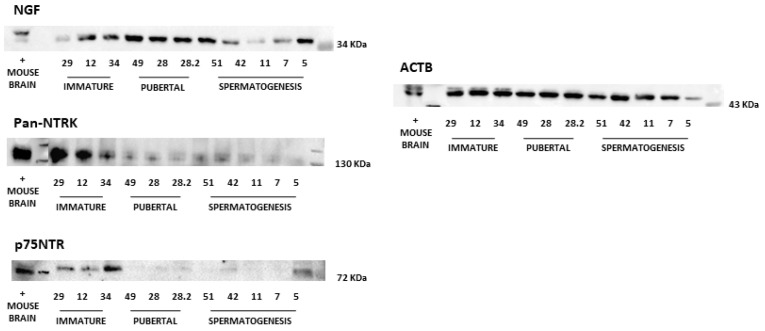
Western blotting of the squirrel testis. Western blotting on the top left is relative to the NGF antibody (kDa 34), the pan-NTRK antibody (kDa 130), and the p75NTR antibody (kDa 72). On the right, the western blot is for the ACTB reference protein. To the left of each western blotting image is shown the positive control (mouse brain) followed by the three sexual phenotypes: immature (lanes 1, 2, and 3), pubertal (lanes 4, 5, 6), and spermatogenesis (lanes 7, 8, 9, 10, and 11). Numbers below the lanes represent the Sample ID. To the right of each western blot, the weight in kDa of the respective antibodies is revealed in the running line outlined in the box.

The Pan-NTRK antibody was directed to a homologous region of NTRK1, TRKB, and TRKC, adjacent to the C-terminus (Figure 3).

**Figure 4:**
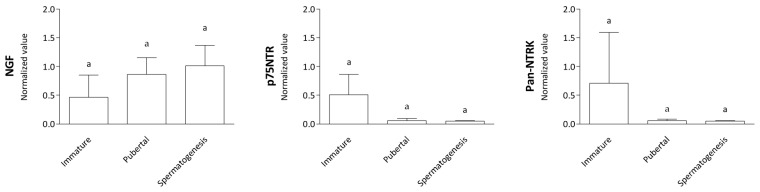
Protein expression-normalized data. The graphs show the results derived by analyzing the western blot images. The expression of the three proteins NGF, p75NTR, and pan-TRK (pan-TRK antibody recognizes the active domains of the neurotrophins NGF, BDNF, and NT-3) is normalized for the value of the reference protein ACTB. Statistical analysis shows no significant differences in NGF, pan-NTRK, and p75NTR protein levels of the immature, pubertal, and spermatogenesis groups. Different letters placed on the top of the boxes indicate statistically significant differences.

Normalized NGF, pan-NTRK, and p75NTR, protein expression showed no difference (*p* > 0.05) between the immature, pubertal, and spermatogenesis groups. Although the statistical analysis did not reveal significant differences in the protein levels of p75NTR and NTRK1 across reproductive stages, visual inspection of the western blot images suggests a decrease in signal intensity during the pubertal and spermatogenesis phases. This observation may indicate a potential modulation of receptor expression to reproductive maturity (Figure 4).

### 3.4. Immunohistochemistry Protein Localization of NGF and NGF Receptors

Immunohistochemical analyses highlighted the presence of the NGF, NTRK1, and p75NTR on all grey squirrel testis samples examined with different localization in the three morphotypes.

**Figure 5:**
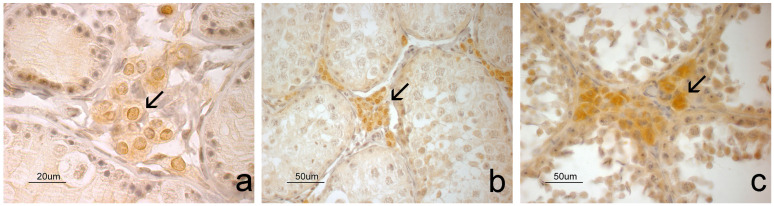
NGF immunopositivity in grey squirrel testis morphotypes. (**a**–**c**) NGF protein is observed at the level of Leydig cells (arrows). The positivity appears more intense in the pubertal morphotype (**b**) and active spermatogenesis (**c**) compared to the immature morphotype (**a**).

The NGF molecule was observed on the Leydig cells, few cells appeared in the immature testes, and progressively more numerous and intensely marked cells appeared in the testes of animals in puberty and subjects undergoing active spermatogenesis, respectively (Figure 5).

**Figure 6:**
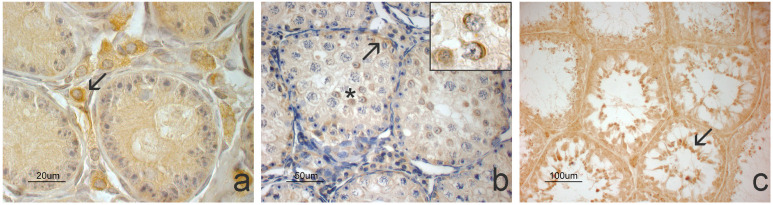
Immunopositivity for NTRK1 in grey squirrel testis morphotypes. The receptor is shown in Leydig cells of the immature morphotype ((**a**), arrow); in basal germ cells (arrow) and in the perinuclear region of type I spermatocytes (asterisk and top panel) of the pubertal morphotype (**b**); and in spermatids and spermatozoa of the mature morphotype ((**c**), arrow).

NTRK1 was observed in Leydig cells in the immature morphotype. In the pubertal morphotype, a weak positivity was observed in the germ cells located in the basal compartment of the epithelium and the perinuclear regions of type I spermatocytes. In the mature morphotype, NTRK1 was localized in germ cells with greater intensity in the spermatids and spermatozoa (Figure 6).

**Figure 7:**
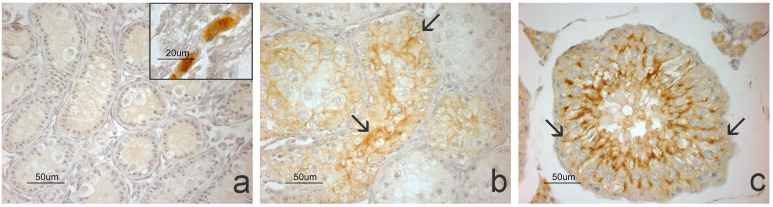
Immunopositivity for p75NTR in the morphotypes of grey squirrel testes. p75NTR is not visible in the parenchyma of the immature morphotype (**a**) where the nerves located in the capsule (top panel) appear positive, providing validity to the immunohistochemical reaction. In the pubertal morphotype (**b**) a weak positivity of Sertoli cells is observed (arrows) which significantly increases in the mature morphotype ((**c**), arrow).

p75NGF was not observed in the immature testicular parenchyma, where receptor-positive nerve bundles were observed representing the positive control internal to the section. The positivity to the receptor emerged, albeit weakly, in the seminiferous tubules of the testis pubertal phase and appeared strong in subjects in active spermatogenesis (Figure 7).

### 3.5. NGF Protein Plasma Levels

**Figure 8:**
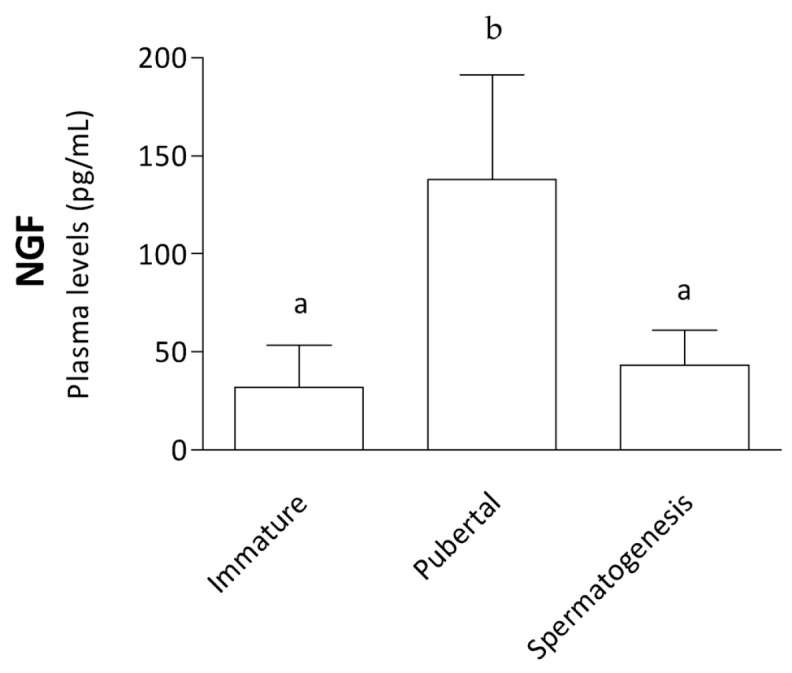
NGF protein plasma content. The graph depicts NGF levels assessed by the ELISA method in the plasma of immature, pubertal, and spermatogenesis stages. In the pubertal group, the level of NGF is higher than the two other groups (*p* ˂ 0.01). Different letters placed on the top of the boxes indicate statistically significant differences.

Plasma NGF levels as determined by the ELISA method showed a higher concentration (*p* ˂ 0.01) in pubertal squirrels (135.80 ± 12 pg/mL) compared with immature (25.60 ± 9.32 pg/mL) and spermatogenesis individuals (34.20 ± 6.06 pg/mL) (Figure 8).

## 4. Discussion

It is well-established that the NGF molecule plays a leading role in the development and regulation of the male reproductive system, both during the individual’s development and in adulthood [10]. NGF’s action has been studied in various animal species, not only at the level of the gonads but also in the genital tract, such as the epididymis and vas deferens [36], as well as in accessory glands like the seminal vesicles, prostate, and bulbourethral glands [6]. Although the role of NGF in testis function has been extensively studied in many species [37,38], this is the first study on the role of NGF and its receptors in the testis function of squirrels across different reproductive phases (immature, pubertal, and active spermatogenesis). NGF and its receptors are likely involved in the various reproductive stages of testis development and maturation. This system has a pivotal role in controlling the reproductive function of this species, in addition to its well-known regulatory role in the nervous system. In this research, the reproductive role of NGF was investigated using different methods (western blotting, qPCR, immunohistochemistry, and ELISA), which together helped identify the presence of the protein and transcript and localized the molecules within the testis tissue. The normalized western blotting results revealed no significant differences between the three reproductive phases in the protein levels of NGF, p75NTR, and pan-TRK. Although the normalized western blot data did not reveal statistically significant differences in protein levels of p75NTR and pan-TRK across the reproductive phases, the observed decrease in signal intensity during the pubertal and spermatogenesis stages suggests a potential biological modulation of the NGF system in these phases. This reduction, while not reaching statistical significance, may reflect underlying physiological changes associated with reproductive development and function in the grey squirrel. The presence of NGF in the testes of grey squirrels aligns with its known expression in the testes of various domestic (llamas [13], rabbits [12], and bulls [6,39]), and wild species [15], indicating that NGF may play a regulatory role in the reproductive processes of grey squirrels. *NGF* gene expression in grey squirrel testes at different stages of sexual maturity revealed an increase in the NGF transcript during puberty. No significant variation was detected between the different testicular morphotypes for the transcripts of the two analyzed receptors. The testicular expression of the NGF system, as measured by qPCR, showed a similar pattern to the plasma NGF concentrations across reproductive phases. This underscores how the testes are an important site of production of NGF, and developing testes probably contribute to the higher levels evaluated in these animals. The circulating NGF results showed a different trend from the western blotting testis protein ones, probably due to the circulating NGF being absorbed by many different tissues. Through an immunohistochemical investigation, NGF was observed in Leydig cells in all testicular morphotypes with an intensity of immunostaining greater in pubertal subjects compared to immature and mature ones. The greater coloration was likely an indication of greater protein production [32,40,41,42]. The difference observed between morphotypes was partially confirmed by the evaluation carried out with qPCR where the quantity of NGF transcript appeared significantly higher in pubertal subjects compared to immature subjects and in active spermatogenesis. The increased expression of ligands around puberty suggests that this molecule can play a role in stimulating the development of testicular parenchyma. As highlighted by the morphological evaluation, in this period important morphological changes were observed in the seminiferous tubules which, in addition to increasing in diameter, showed the formation of the lumen and the development of the germ cell population [27].

The role of NGF in the male reproductive system was demonstrated during both testicular morphogenesis and the regulation of spermiogenesis, as evidenced by the presence of NGF receptors in Sertoli cells [43,44]. Further evidence includes lower NGF levels in infertile individuals [45]. NGF plays a critical role in maintaining the physiological integrity of the cells of the seminiferous epithelium [46], stimulating DNA synthesis in the seminiferous tubules [47] and inducing the secretion of androgen-binding protein (ABP) from Sertoli cells [48].

The detection of p75NTR and NTRK1 receptors within the testicular parenchyma suggests that NGF may act through paracrine and/or autocrine signaling in the squirrel testis. As evidence of this, in vitro studies on various cell lines have demonstrated that NGF can function as a local signaling factor, facilitating interactions between somatic and spermatogenic cells [49]. In this study, the NGF receptor was observed in the testis of Umbrian grey squirrels, with increasing immunostaining intensity in the Sertoli cells of pubertal and mature morphotypes, while no positivity was detected in the immature morphotype. These data suggest that the NGF molecule can act on the immature testis via the NTRK1 localized in the Leydig cells. From puberty and even later, that is in the full scope of reproductive activity, the NGF system appeared complete, indicating that the ligand could activate both the receptors that operate synergistically and competitively [50]. Through qPCR, the NGF receptors p75NTR and NTRK1 were detected in immature squirrels as well as in pubescent and mature ones. The discrepancy between the qPCR and immunohistochemistry could be due to the greater sensitivity of the first method in detecting the presence of transcripts; on the other hand, in this period of development, the NGF receptors protein could still be poorly concentrated due to limited translation and accordingly not being visible in a morphological observation. NTRK1 showed variable cellular localization based on the grey squirrel testis morphotype analyzed. Differences in the localization of NGF and related receptors at different periods of testis development have also been observed in other animal species [43,51]. Jin et al. (2010) [52] hypothesized a role of NGF in the maturation of spermatids by observing NGF during the different phases of spermiogenesis in the golden hamster; it could also be valid for the grey squirrel, as NTRK1 was observed in the germ cells of pubescent and mature subjects.

The immunohistochemical results obtained in this study for NGF and its two receptors find only partial confirmation from the numerous and conflicting data already present in the literature for other rodents and animal species [15,36,46,49,53]. The differences described in the literature relating to the identification, localization, and expression of the molecule and its receptors suggest that the NGF system is species-specific for localization and function, highlighting the complexity of defining the mechanisms that regulate this system in reproductive activity. In addition, it is necessary to consider that the cellular localization of NGF and related receptors could change with development [43,51]. However, the role of NGF in the testis and reproductive processes appears indisputable. The indispensable role of NGF in testicular and reproductive processes is further supported by studies demonstrating its involvement in spermatogenesis, testicular development, and hormonal regulation across species. For example, NGF has been shown to influence the activation of primordial follicles and folliculogenesis in knockout models of mice, highlighting its critical function in reproductive physiology [54]. As previously shown in other mammalian species [10], our findings indicate a potential auto- and/or paracrine pathway through which the NGF system may be involved in grey squirrel reproductive activity.

These findings could inform strategies to control grey squirrel populations by targeting reproductive mechanisms regulated by NGF and its receptors, potentially developing interventions that disrupt reproductive success and limit population growth. With an emphasis on the biological roles of the NGF receptor and the NGF source, future studies should assess the involvement of NGF and its corresponding receptors in the reproductive processes of IAS grey squirrels.

## 5. Conclusions

This study provides novel insights into the role of NGF and its receptors in the reproductive physiology of grey squirrels. By examining NGF system expression and localization across different reproductive phases—immature, pubertal, and active spermatogenesis—our findings reveal that NGF is consistently present in the testes and exhibits significant changes during puberty. The data support the hypothesis that NGF plays a role in testicular development and function, potentially through autocrine or paracrine mechanisms. The observed increase in NGF expression during puberty aligns with morphological changes in the seminiferous tubules, suggesting a regulatory function of NGF in testis maturation. These results not only underscore the importance of NGF in squirrel reproductive biology but also highlight the need for further research to fully elucidate its mechanisms and effects in this and other species.

## Figures and Tables

**Table 1:** QPCR primers. The table shows *NGF*, *NTRK1*, *p75NTR*, and *ACTB* (*Actin B*) forward (F) and reverse (R) primer sequences [31] and expected amplicon (bp).

Gene	NCBI Genebank Accession Number		Primers	bp
*NGF*	XM_015502222.2	F	TCCACCCACCCAGTCTTC	178
		R	GCTCGGCACTTGGTCTCA	
*NTRK1*	XM_047554612.1	F	TCGGACCATGCTGCCCATCC	261
		R	AGGCGTTGCTGCGGTTCTCG	
*p75NTR*	XM_047545416.1	F	GGAGGACACGAGTCCTGAGC	295
		R	CAGTGGAGAGTGCTGCAAAG	
*ACTB*	XM_047552944.1	F	TTGTGATGGACTCCGGAGAC	186
		R	TGATGTCACGCACGATTTCC	

**Table 2:** Antisera characteristics. The table shows the antisera name, the species in which the antibody is raised, the working solution, and the antibody producer.

Antisera	Species	Dilution	Commercialized
NGF Recombinant monoclonal	Rabbit	1:100	MA5-32067, Thermo Fisher Scientific, Waltham, MA, USA
Anti-NTRK1	Rabbit	1:100/1:500	Maranesi et al., 2024 [33]
Anti-p75NTR	Rabbit	1:100	ab52987, Abcam, Cambridge, UK
Anti-rabbit biotin-conjugated	Goat	1:200	BA-1000-1.5, Vector Laboratories, Newark, CA, USA
Rabbit IgG	Rabbit	1:100	I-1000-5, Vector Laboratories, Newark, CA, USA [35]

## Data Availability

The original contributions presented in the study are included in the article, further inquiries can be directed to the corresponding author.
